# Prevalence and Genetic Diversity of *Enterococcus faecalis* Isolates from Mineral Water and Spring Water in China

**DOI:** 10.3389/fmicb.2017.01109

**Published:** 2017-06-16

**Authors:** Lei Wei, Qingping Wu, Jumei Zhang, Weipeng Guo, Moutong Chen, Liang Xue, Juan Wang, Lianying Ma

**Affiliations:** ^1^State Key Laboratory of Applied Microbiology Southern China, Guangdong Provincial Key Laboratory of Microbial Culture Collection and Application, Guangdong Open Laboratory of Applied Microbiology, Guangdong Institute of MicrobiologyGuangzhou, China; ^2^College of Food Science, South China Agricultural UniversityGuangzhou, China

**Keywords:** *Enterococcus faecalis*, mineral water, spring water, ERIC-PCR, virulence genes

## Abstract

*Enterococcus faecalis* is an important opportunistic pathogen which is frequently detected in mineral water and spring water for human consumption and causes human urinary tract infections, endocarditis and neonatal sepsis. The aim of this study was to determine the prevalence, virulence genes, antimicrobial resistance and genetic diversity of *E. faecalis* from mineral water and spring water in China. Of 314 water samples collected from January 2013 to January 2014, 48 samples (15.3%) were contaminated *E. faecalis*. The highest contamination rate occurred in activated carbon filtered water of spring water (34.5%), followed by source water of spring water (32.3%) and source water of mineral water (6.4%). The virulence gene test of 58 *E. faecalis* isolates showed that the detection rates of *asa1*, *ace*, *cylA*, *gelE* and *hyl* were 79.3, 39.7, 0, 100, 0%, respectively. All 58 *E. faecalis* isolates were not resistant to 12 kinds of antibiotics (penicillin, ampicillin, linezolid, quinupristin/dalfopristin, vancomycin, gentamicin, streptomycin, ciprofloxacin, levofloxacin, norfloxacin, nitrofurantoin, and tetracycline). Enterobacterial repetitive intergenic consensus-PCR classified 58 isolates and three reference strains into nine clusters with a similarity of 75%. This study is the first to investigate the prevalence of *E. faecalis* in mineral water and spring water in China. The results of this study suggested that spring water could be potential vehicles for transmission of *E. faecalis*.

## Introduction

Enterococci mainly inhabits in human and animal faces, ham sausage, pasteurized milk and drinking water (du Toit et al., 2000; Franz et al., 2003; Zou et al., 2011). Although some Enterococcal species are considered relevant for their technological properties (such as ripening, aroma development and inhibition of pathogens), they are not, unlike other lactic acid bacteria, recognized as probiotics (Kuriyama et al., 2003; Emaneini et al., 2008; Jamet et al., 2012). Indeed, enterococci are a major cause of nosocomial infections, such as urinary tract infections, endocarditis and neonatal sepsis (Franz et al., 2011; Sanchez Valenzuela et al., 2012; Werner et al., 2013). The main enterococcal isolates involved in nosocomial infections is *Enterococcus faecalis* (Giraffa, 2002; Kayser, 2003). *E. faecalis* is an important opportunistic pathogen, which is frequently detected in mineral water and spring water for human consumption (Nicas et al., 1989; Martin-Platero et al., 2009; Buhnik-Rosenblau et al., 2013). Several studies have indicated that *E. faecalis* is a suitable indicator of the presence of pathogens in mineral water and spring water (Švec and Sedláček, 1999; Davis et al., 2005; Meng, 2007). The Natural Mineral Water National Standard GB 8537 (the National Food safety Standards of China) has short-listed *E. faecalis* as a microorganism indicator in mineral water and spring water factory in China.

For surveillance or tracing sources of *E. faecalis*, molecular typing methods such as pulsed field gel electrophoresis (PFGE) (Weng et al., 2013), amplified fragment length polymorphism (AFLP) (Bruinsma et al., 2002), random amplified polymorphic DNA (RAPD) (Martin-Platero et al., 2009), multi-locus sequence typing (MLST) (Homan et al., 2002; Werner et al., 2012) and enterobacterial repetitive intergenic consensus (ERIC-PCR) (Martin-Platero et al., 2009), can be used in *E. faecalis* isolates. Among these molecular typing approaches, PFGE is the most effective technology or typing of *E. faecalis* isolates due to its high reproducibility and discriminatory ability. However, PFGE is labor intensive and time-consuming (Weng et al., 2013). In contrast, ERIC-PCR is a relatively simple and cost-effective method, which has been successfully used for genotyping of different bacterial pathogens and for tracking the bacterial source of contaminated water products (Martin-Platero et al., 2009).

*Enterococcus faecalis* can produce dozens of virulence substances including hemolysin and surface adhesion substances (Aslam et al., 2012; Choi and Woo, 2013). The pathogenesis of five virulence genes including *asa1*, *ace*, *cylA*, *gelE* and *hyl* has been systematically studied. The *asa1*gene encodes surface adhesion substances by which *E. faecalis* can be fixed on a eukaryotic cell; the *ace* gene encodes surface adhesion proteins by which *E. faecalis* are resistant to immune function of the host cells; the *cylA* gene encodes hemolysin which can leads to the death of host cells; the *gelE* gene encodes gelatinase, which can hydrolyze gelatin leading to the proliferation of bacteria in the host cell; the *hyl* gene encodes hyaluronidase which can hydrolyze the tissue of host cell (Coque et al., 1995; Eaton and Gasson, 2001; Cosentino et al., 2010; Yang et al., 2015).

**FIGURE 1:**
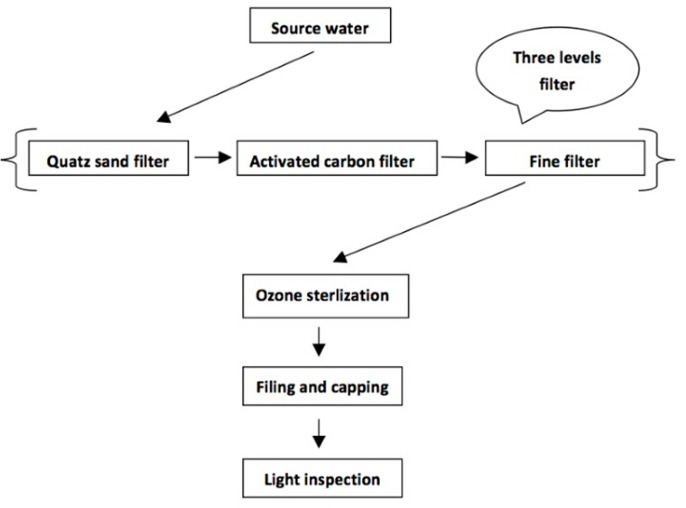
Production flow chart of mineral water and spring water in China.

As shown in **Figure 1**, mineral water and spring water are generally produced using the same process, including three level filter (quartz sand filter, activated carbon filter, and fine filter), ozone sterilization, filling and capping, and light inspection of finished product. For manufacturers, the quality of source water, activated carbon filtered water and finished product are important. Source water reflects raw quality of mineral and spring water, and finished product is for human consumption. Activated carbon filter with a lot of pores and large surface area is a kind of common water treatment equipment, which processes a strong physical adsorption capacity to absorb organic pollutants and microbes. Recent studies have shown that activated carbon filter has become a gathering place for microbes and is the most serious in microbial contamination in whole production process of mineral water and spring water.

Systematic contamination survey of *E. faecalis* in mineral water and spring water has not yet been conducted. The aim of this study was for the first time to determine the prevalence, virulence genes, antimicrobial resistance and genetic diversity of *E. faecalis* from mineral water and spring water in China. The information generated in this study will provide insights into the prevalence and differentiation of *E. faecalis* isolates in mineral water and spring water.

## Materials and Methods

### Sample Collection

From January 2013 to January 2014, a total of 314 water Samples were collected from 101 mineral water and spring water factories in 10 provinces of China (Guangdong, Guangxi, Fujian, Hainan, Hubei, Shanghai, Beijing, Yunnan, Guizhou and Sichuan). Samples of source water (112), activated carbon filtered water (101) and finished product (101) of spring water and mineral water were collected from each water factories. Samples of source water (112) include Surface water (14) and Groundwater (98). All water samples were maintained at 4°C during transportation and testing was conducted within 1 h after receiving the samples.

### Isolation and Enumeration of *E. faecalis*

Briefly, 250 ml of water sample was filtered through a 0.45 μm membrane (Millipore Co., Billerica, MA, United States) in a stainless steel multi-line filter system (Huankai Co., Guangzhou, China). The membrane was placed on KF agar medium (Huankai Co., Guangzhou, China), a selective medium for *E. faecalis* and then cultured at 36°C for 2 days. Presumptive colonies with red color were selected for catalase test and cultured in brain heart infusion broth at 45°C for 2 days and Bile broth at 36°C for 3 days, respectively. Colonies positive for the three confirmation tests were considered presumed *E. faecalis*.

### *Enterococcus faecalis* Identification

All the presumed *E. faecalis* were identified by the API 20 STREP biochemical identification system (Biomerieux Co., Lyon, France) and specific PCR for the *E. faecalis* species. According to seven code of API 20 Strep biochemical identification system, coincidence rate of *E. faecalis* isolates can be tested. Further identification of *E. faecalis* was determined by PCR using *sodA* species-specific primers. Genomic DNA was extracted from collected *E. faecalis* isolates by using a Bacterial Genomic DNA Purification kit (Dongsheng Biotech, Guangzhou, China) according to the manufacturer’s instruction. *E. faecalis* were identified by amplification of 210 bp fragments with primer pairs EFS1 (5′ CTGTAGAAGACCTAATTTCA)/EFS2 (5′ CAGCTGTTTTGA AAGCAG) (Jamet et al., 2012).

### ERIC-PCR Analysis

Total DNA from *E. faecalis* isolates was extracted as previously described. Genomic DNA concentration was determined at 260 nm by using a Nano Drop ND 1000 UVe-Vis spectrophotometer (Thermo Fisher Scientific, Waltham, MA, United States). The ERIC primers used were referred from the study of Versalovic et al. (1991). ERIC-PCR typing was performed for the collected *E. faecalis* isolates by using the protocol described by Li (2013) with some modifications. The PCR mixture (25 μl) contained 1.5 unit Hot start polymerase (Promega, Madison, WI, United States), 0.5 μmol/l each primer, 2.5 μmol/l MgCl_2_, 200 μmol/l each dNTP, and 1 μl of the template genomic DNA (50 ng). Amplifications were performed with a DNA thermocycler (Applied Biosystems, Foster City, CA, United States) under the following temperature profiles: an initial denaturation at 95°C for 5 min; 35 cycles of 1 min at 94°C, 1 min at 36°C and 2 min at 72°C; and a final extension at 72°C for 8 min (Martin-Platero et al., 2009; Li, 2013). The ERIC-PCR products were separated by electrophoresis in a 1.5% agarose gel with Goldview staining (0.005%, v/v) and then photographed using a UV Imaging System (GE Healthcare, Milwaukee, WI, United States). The images were captured in TIFF file format for further analysis.

### Detection of Virulence Genes

Five virulence genes, *asa1*, *ace*, *cylA*, *gelE* and *hyl* were individually detected in all the collected *E. faecalis* isolates with the PCR technique (Eaton and Gasson, 2001; Zou et al., 2011). All primers were synthesized by Sangon Biotech company (Shanghai, China). The primers used to identify virulence genes are shown in **Table 1**. *E. faecalis* isolates CMCC 32219 (Guangdong culture collection centre) was used as positive control and distilled water was used as the negative control.

**Table 1:** PCR primers for virulence gene detection.

No.	Primers	Sequence (5’→3’)	bp	Function
1	asa1-F	CACGCTATTACGAACTATGA	375	Surface adhesion
	asa1-R	TAAGAAAGAACATCACCACGA		substances
2	ace-F	GGAATGACCGAGAACGATGGC	616	Surface adhesion
	ace-R	GCTTGATGTTGGCCTGCTTCCG		proteins
3	cylA-F	ACTCGGGGATTGATAGGC	688	Hemolysin
	cylA-R	GCTGCTAAAGCTGCGCTT		
4	gelE-F	TATGACAATGCTTTTTGGGAT	213	Gelatinase
	gelE-R	AGATGCACCCGAAATAATATA		
5	hyl-F	ACAGAAGAGCTGCAGGAAATG	276	Hyaluronidase
	hyl-R	GACTGACGTCCAAGTTTCCAA		

### Antibiotic Resistance

According to the Clinical and Laboratory Standards Institute (CLSI) standards, all the collected *E. faecalis* isolates were tested by the disk diffusion method (Clinical and Laboratory Standards Institute [CLSI] (2006)). *E. faecalis* isolates CMCC 32219 (Guangdong culture collection centre) was used as positive control. A panel of antibiotics at the specific concentration per disk were tested: penicillin G (10 U), ampicillin (10 μg), linezolid (30 μg), quinupristin/dalfopristin (15 μg), vancomycin (30 μg), gentamicin (120 μg), streptomycin (10 μg), ciprofloxacin (5 μg) levofloxacin (5 μg), norfloxacin (10 μg), nitrofurantoin (300 μg), tetracycline (30 μg) (Oxoid Co., Hampshire, United Kingdom) (Jamet et al., 2012; Sanchez Valenzuela et al., 2013). The isolates were classified as sensitive, intermediate, and resistant using the breakpoints specified by the CLSI.

### Fingerprint Data Analysis

ERIC-PCR fingerprint patterns were analyzed using a Gel-Pro analyser (version 6.0) according to the manufacturer’s instructions. The observed bands in the gels were evaluated based on the presence (code 1) or absence (code 0) of polymorphic fragments for the ERIC products. The cluster analysis was performed using NTSYSpc (version 2.10e), and similarities between ERIC-PCR profiles were calculated based on the simple-matching similarity matrix and unweighted pair group method with arithmetic.

## Results

### Contamination of *E. faecalis* in Mineral Water and Spring Water

Of the 314 water samples tested, 48 (15.3%) were positive for *E. faecalis*, including 24 (21.4%) of 112 source water, 22 (21.8%) of 101 activated carbon filtered water samples and 2 (1.9%) of 101 finished product samples. From the contaminated samples, 58 *E. faecalis* isolates were obtained (Supplementary Table S1). As shown in **Table 2**, the rate of *E. faecalis* contamination in all mineral water samples was 3.8%, and the contamination rates of source water, activated carbon filtered water and finished product were 6.4, 4.7, and 0%, respectively. The rate of *E. faecalis* contamination in all spring water samples was 23.8%, and the contamination rates of source water, activated carbon filtered water and finished product were 32.3, 34.5, and 7.5%, respectively. In all the 48 contaminated samples, the contamination levels in spring water samples are significantly higher than those in mineral water samples. The contamination level of source water and activated carbon filtered water were 48.0 and 26.4 CFU/250 ml in spring water samples.

**Table 2:** Prevalence of *E. faecalis* from mineral water and spring water in China.

	Source water		Activated carbon filtered water		Finish product		Total	

**Samples**	**CR (%)**	**CL (CFU/250 ml)**	**CR (%)**	**CL (CFU/250 ml)**	**CR (%)**	**CL (CFU/250 ml)**	**CR (%)**	**CL (CFU/250 ml)**
M water	6.4	2.3	4.7	3.5	0	0	3.8	2.8
S water	32.3	48.0	34.5	26.4	3.4	7.5	23.8	36.0
Average	21.4	42.2	21.8	24.3	1.9	7.5	15.3	32.5

*M and S water represent mineral and spring water, respectively; CR and CL represent contamination rate and level in positive samples, respectively*.

All the 112 source water Samples include 14 surface source water samples and 98 underground source water samples. As shown in **Table 3**, 16 (16.3%) samples of underground source water were positive in all 98 samples. Meanwhile, eight (57.1%) samples of surface source water were positive in all 14 samples.

**Table 3:** Prevalence of *E. faecalis* in surface water and groundwater.

Samples	Positive amounts	Total amounts	Contamination rates%
Surface water	8	14	57.1
Groundwater	16	98	16.3

### Detection of Virulence Genes in *E. faecalis* Isolates

In this study, the presence of *asa1*, *ace*, *cylA*, *gelE* and *hyl* genes was detected in 58 *E. faecalis* isolates. As shown in **Table 4**, all the 58 *E. faecalis* isolates (100%) harbored the *gelE* gene, among which 46 (79.3%) and 23 (39.7%) also had the *asa1* and *ace* genes, respectively. However, no *cylA* or *hyl* gene was detected from all the isolates.

**Table 4:** Virulence genes of 58 *E. faecalis* isolates.

Virulence gene	No. of positive sample (%)
*asa1*	46 (79.3)
*ace*	23 (39.7)
*cylA*	0 (0)
*gelE*	58 (58)
*hyl*	0 (0)

### Antibiotic Resistance

According the diameter of zone of inhibition, all the 58 *E. faecalis* isolates were classified as sensitive, intermediate, and resistant using the breakpoints specified by the CLSI. The result of antibiotic resistance showed that all 58 isolates were sensitive to 12 kinds of antibiotic (penicillin, ampicillin, linezolid, quinupristin/dalfopristin, vancomycin, gentamicin, streptomycin, ciprofloxacin levofloxacin, norfloxacin, nitrofurantoin, and tetracycline) which were selected according to the standard of CLSI. No resistant *E. faecalis* isolate was found.

### Biochemical Identification

With *E. faecalis* ATCC 29212 (a), CMCC 32219 (b) and CMCC 32223 (c) (Guangdong culture collection centre) as positive controls, according to seven code of API 20 Strep biochemical identification system, biotypes of *E. faecalis* isolates can be tested. As shown in **Table 5**, biotypes of 44 *E. faecalis* isolates and two control isolates are 5143711. Biotypes of four *E. faecalis* isolates and one control isolate are 5153711, and biotypes of ten *E. faecalis* isolates are 7143711.

**Table 5:** Biochemical profiles of 58 *E. faecalis* isolates.

Biotypes	No. of isolates	7 code
A	a, b, 2∼5, 8∼11, 14∼36, 38∼41, 44, 46, 49∼52, 54, 56, 58	5143711
B	1, 6∼7, 37, 45, 47∼48, 53, 55, 57	7143711
C	c, 12∼13, 42∼43	5153711

*a, b and c represent *E. faecalis* ATCC 29212, CMCC 32219 and CMCC 3222, respectively*.

### ERIC-PCR

**FIGURE 2:**
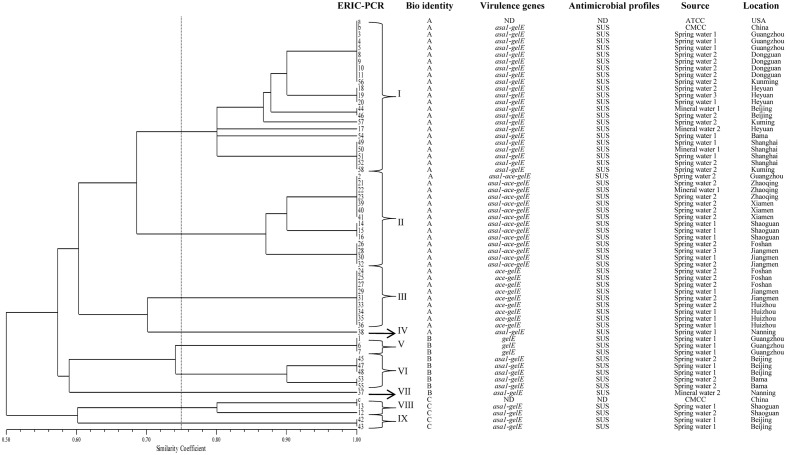
ERIC-PCR DNA fingerprint analysis of *E. faecalis* isolates from mineral water and spring water in China. a, b and c represent *E. faecalis* ATCC 29212, CMCC 32219 and CMCC 32223, respectively; ND, not detect; SUS, susceptibility; 1, 2 and 3 represent source water, activated carbon filtered water and finish product, respectively.

The ERIC-PCR patterns of three control isolates (a, b, c) and 58 collected *E. faecalis* isolates are shown in **Supplementary Figure S1**. The ERIC of DNA profiles consisting of 5 to 15 bands ranged from 230 bp to approximately 4,000 bp. As shown in **Figure 2**, three control isolates (a, b, c) and 58 collected *E. faecalis* isolates were grouped into nine clusters at similarity coefficient of 75%. Control isolates ATCC 29212 (a), CMCC 32219 (b) and 21 collected *E. faecalis* were distributed among the cluster I, a prevalent cluster. Control isolate CMCC 32223 (c) and two collected *E. faecalis* isolates were distributed among cluster VIII. Cluster IV has only one isolate (NO.38) and Cluster VII has only one isolate (NO. 37).

## Discussion

In accordance with the Natural Mineral Water National Standard GB 8537, *E. faecalis* must be absent from 250 ml of water samples. In this study, 48 (15.3%) *E. faecalis*-positive samples from a total of 314 water samples were found. This result was consistent with previous investigations conducted in China (17.0%) (Li, 2013) and Greece (17.4%) (Grammenou et al., 2006). *E. faecalis* has not been systematically studied from mineral water and spring water, and our results were obtained from a large number of samples in China. Therefore, these nationwide data are more beneficial for risk assessment. The *E. faecalis* contamination rates in spring water samples were significantly higher than those in mineral water samples. The high prevalence of *E. faecalis* in the spring water indicated poor hygiene practices during manufacture process. The finished product of spring water presented a contamination rate of 3.4%, which can adversely affect the health of costumers. Therefore, the Chinese food safety management should implement further supervision for spring water products as well as implementation of Good Hygiene Practices (GHP). In addition, contamination rate of source water in surface was significantly higher than those in groundwater. Based on the contamination rate of *E. faecalis*, groundwater is better than surface water as source water.

In this study, *E. faecalis* contamination rate of activated carbon filtered in spring water reached 26.4%, which was the highest among all water samples tested. Activated carbon filter system is commonly used for mineral water treatment to ensure equipment life, improve water quality and prevent pollution. Activated carbon filter which has a lot of pores and large surface area processes a strong physical adsorption capacity to absorb organic pollutants and microbes (Feng et al., 2013). Recent studies have shown that activated carbon filter has become a gathering place for microbes and is the most serious in microbial contamination in whole production process of mineral water and spring water (Camper et al., 1986). Hence, manufacturers of mineral water and spring water must establish measures for monitoring the activated carbon filtration system, as well as ensure timely cleaning and regular replacement of activated carbon (da Silva Fernandes et al., 2015; Fernandes et al., 2015).

Highly virulent *E. faecalis* isolates may cause diseases even at relatively low concentrations. In this study, up to 100% of *E. faecalis* isolates were *gelE*-positive and 79.3% were *asa1*-positive, which is consistent with previous studies and provides further evidence that these virulence genes are widely distributed among *E. faecalis* (Moraes et al., 2012; Anderson et al., 2015). The presence of these genes indicates pathogenic potential and probability to cause diseases. However, the expression of virulence genes is mainly related to quorum sensing. Further studies must be performed to examine whether the *E. faecalis* isolates in this study are pathogenic (Huebner et al., 1999; Delpech et al., 2012). All the 58 collected *E. faecalis* isolates were *cylA*-negative and *hyl*-negative, which is not consistent with previous studies (Creti et al., 2004; Medeiros et al., 2014). All the 58 collected *E. faecalis* isolates were sensitive to 12 kinds of antibiotic, which is different from previous investigation about drink water (Macedo et al., 2011), food (Gaglio et al., 2016) and clinical samples (Dahlén et al., 2012; Gilmore et al., 2013). The difference of the results may be due to different sources of the samples. The antibiotic resistance of *E. faecalis* come from different sources have huge difference (Abriouel et al., 2008). In this study, most of collected *E. faecalis* isolates derived from groundwater. So far, there is almost no research on antibiotic resistance of *E. faecalis* isolated from the groundwater.

ERIC-PCR is one of the most widely adopted PCR typing methods and is chosen for analyses of genetic diversity (Chen et al., 2014; Xie et al., 2015; Zhang et al., 2015). This method provides discriminatory value and is a rapid method for *E. faecalis* typing. In this study, the ERIC-PCR results provided a better overview of *E. faecalis* diversity. Most of collected *E. faecalis* isolates in the same area belong to the same cluster, which agrees with the results of previous studies (Martin-Platero et al., 2009). Three isolates (18, 19 and 20) obtained from spring water in Heyuan city showed 100% similarity. Two isolates (44 and 46) from spring water in Beijing city also showed identical ERIC patterns. Additionally, 10 isolates (a, b, 3, 4, 5, 8, 9, 10, 11 and 58) from different sources yielded an identical pattern, suggesting that they were highly homogenous and had a close genetic relationship. A correlation between the genomic profiles and the virulence genes was observed in these strains. Most of the isolates that carried *asa1*, *ace* and *gelE* were grouped in cluster B. In this cluster, a good correlation among ERIC patterns, virulence profiles, and the sample source was found in some isolates. In this study, no antibiotic resistance isolate was found, so no correlation was observed between the ERIC-PCR profiles and the antibiotic resistance profiles of the isolates.

In summary, our study for the first time revealed the high prevalence of *E. faecalis* from mineral water and spring water in China, which should have a potentially pathogenic effect on the health of consumers. The results of this study suggested that spring water product could be potential vehicles for transmission of *E. faecalis*. Meanwhile mineral water and spring water manufacturing factories must pay high attention to the contamination of activate carbon filters. These data may provide useful information for the development of public health policies and effective strategies to ensure the safety of our drinking water products.

## Ethics Statement

All procedures performed in studies involving human participants were in accordance with the ethical standards.

## Author Contributions

Conceived and designed the experiments: QW, JZ, and LW. Performed the experiments: LW and WG. Analyzed the data: LW, MC, and LX. Contributed reagents/materials/analysis tools: JW and LM. Contributed to the writing of the manuscript: LWand QW.

## Conflict of Interest Statement

The authors declare that the research was conducted in the absence of any commercial or financial relationships that could be construed as a potential conflict of interest.

## Supplementary Material

FIGURE S1 Dendrogram of ERIC-PCR patterns of collected *E. faecalis* isolates. M and CK represents marker and control check, respectively; a, b and c represent *E. faecalis* ATCC 29212, CMCC 32219 and CMCC 3222, respectively.
