# Supplementary material for: Prevalence and Genetic Diversity of Enterococcus faecalis Isolates from Mineral Water and Spring Water in China
Source: Front Microbiol. 2017 Jun 16;8:1109. doi: 10.3389/fmicb.2017.01109 (PMC5472655; doi:10.3389/fmicb.2017.01109)
Supplement: FIGURE S1 — Dendrogram of ERIC-PCR patterns of collected E. faecalis isolates. M and CK represents marker and control check, respectively; a, b and c represent E. faecalis ATCC 29212, CMCC 32219 and CMCC 3222, respectively. [file Data_Sheet_1.DOCX]

Supplementary Material

Prevalence and genetic diversity of *Enterococcus faecalis* isolates from mineral water and spring water in China

**Lei WEI^1^, Qingping WU^1^*, Jumei ZHANG^1^, Weipen GUO^1^, Moutong CHEN^1^, Liang XUE^1^, Juan WANG^1, 2^, Lianying MA^1^**

*** Correspondence:** Qingping Wu: E-mail: [wuqp203@163.com](mailto:wuqp203@163.com)

## Supplementary Table

Tabel S1 Information of *enterococcus faecalis isolates*

| Isolates No. | Sample No. | source | Location |
| --- | --- | --- | --- |
| 1 | GZ-S1-1 | Source water | Guangzhou |
| 2 | GZ-S1-2 | Activated carbon filtered water | Guangzhou |
| 3 | GZ-S2-1 | Source water | Guangzhou |
| 4 | GZ-S2-1 | Source water | Guangzhou |
| 5 | GZ-S3-1 | Source water | Guangzhou |
| 6 | GZ-S4-1 | Source water | Guangzhou |
| 7 | GZ-S5-1 | Source water | Guangzhou |
| 8 | DG-S1-2 | Activated carbon filtered water | Dongguang |
| 9 | DG-S2-2 | Activated carbon filtered water | Dongguang |
| 10 | DG-S3-2 | Activated carbon filtered water | Dongguang |
| 11 | DG-S4-2 | Activated carbon filtered water | Dongguang |
| 12 | SG-S1-1 | Source water | Shaoguan |
| 13 | SG-S1-1 | Source water | Shaoguan |
| 14 | SG-S2-1 | Source water | Shaoguan |
| 15 | SG-S2-1 | Source water | Shaoguan |
| 16 | SG-S2-2 | Activated carbon filtered water | Shaoguan |
| 17 | HY-M1-2 | Activated carbon filtered water | Heyuan |
| 18 | HY-S1-2 | Activated carbon filtered water | Heyuan |
| 19 | HY-S1-3 | Finish product | Heyuan |
| 20 | HY-S1-1 | Source water | Heyuan |
| 21 | ZQ-S1-2 | Activated carbon filtered water | Zhaoqing |
| 22 | ZQ-M1-1 | Source water | Zhaoqing |
| 23 | ZQ-S1-2 | Activated carbon filtered water | Zhaoqing |
| 24 | FS-S1-2 | Activated carbon filtered water | Foshang |
| 25 | FS-S1-2 | Activated carbon filtered water | Foshang |
| 26 | FS-S2-2 | Activated carbon filtered water | Foshang |
| 27 | FS-S3-2 | Activated carbon filtered water | Foshang |
| 28 | JM-S1-3 | Finish product | Jiangmen |
| 29 | JM-S1-1 | Source water | Jiangmen |
| 30 | JM-S2-1 | Source water | Jiangmen |
| 31 | JM-S1-2 | Activated carbon filtered water | Jiangmen |
| 32 | JM-S1-2 | Activated carbon filtered water | Jiangmen |
| 33 | HZ-S1-2 | Source water | Huizhou |
| 34 | HZ-S1-1 | Source water | Huizhou |
| 35 | HZ-S2-1 | Source water | Huizhou |
| 36 | HZ-S2-1 | Source water | Huizhou |
| 37 | NN-M1-2 | Activated carbon filtered water | Nanning |
| 38 | NN-S1-1 | Source water | Nanning |
| 39 | XM-S1-2 | Activated carbon filtered water | Xiamen |
| 40 | XM-S1-2 | Activated carbon filtered water | Xiamen |
| 41 | XM-S2-2 | Activated carbon filtered water | Xiamen |
| 42 | BJ-S1-1 | Source water | Beijing |
| 43 | BJ-S2-1 | Source water | Beijing |
| 44 | BJ-M1-1 | Source water | Beijing |
| 45 | BJ-S3-2 | Activated carbon filtered water | Beijing |
| 46 | BJ-S1-2 | Activated carbon filtered water | Beijing |
| 47 | BJ-S3-1 | Source water | Beijing |
| 48 | BJ-S4-1 | Source water | Beijing |
| 49 | SH-S1-1 | Source water | Shanghai |
| 50 | SH-M1-1 | Source water | Shanghai |
| 51 | SH-S2-1 | Source water | Shanghai |
| 52 | SH-S1-2 | Activated carbon filtered water | Shanghai |
| 53 | BM-S1-2 | Activated carbon filtered water | Bama |
| 54 | BM-S1-1 | Source water | Bama |
| 55 | BM-S1-2 | Activated carbon filtered water | Bama |
| 56 | KM-S1-2 | Activated carbon filtered water | Kunming |
| 57 | KM-S1-2 | Activated carbon filtered water | Kunming |
| 58 | KM-S2-2 | Activated carbon filtered water | Kunming |

## Supplementary Figures


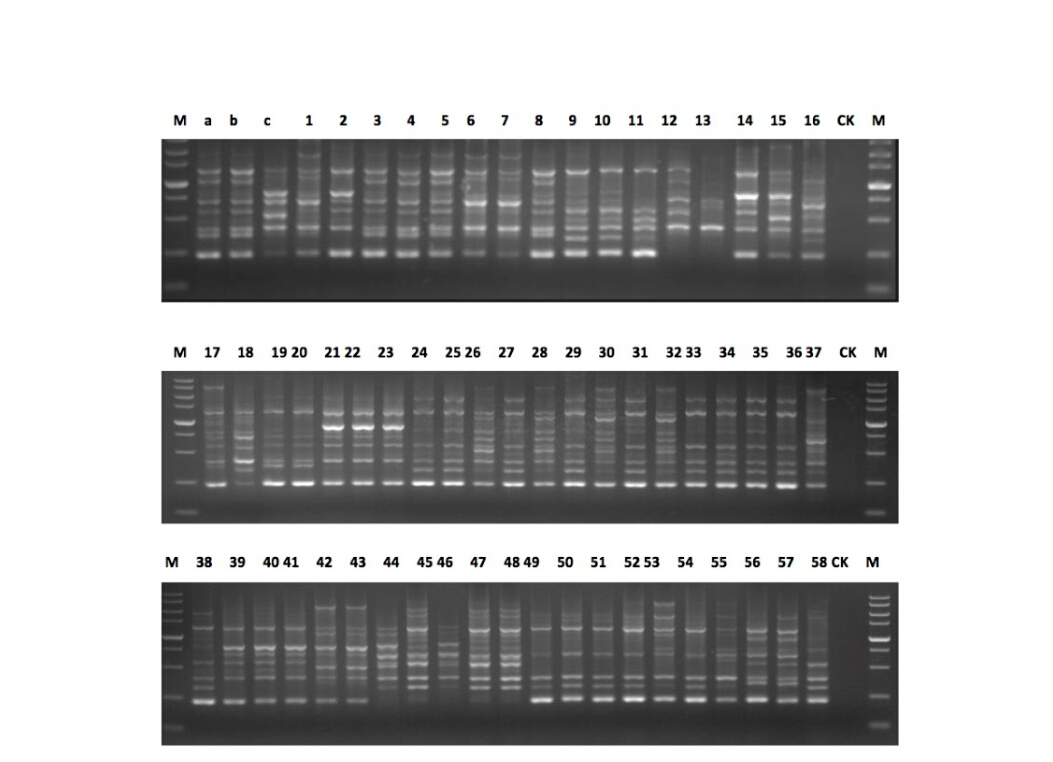


Fig. S1 ERIC-PCR patterns of *E. faecalis*
